# Cognitive behavioral therapy for overactive bladder in women: study protocol for a randomized controlled trial

**DOI:** 10.1186/s12894-020-00697-0

**Published:** 2020-08-20

**Authors:** Satoshi Funada, Norio Watanabe, Takayuki Goto, Hiromitsu Negoro, Shusuke Akamatsu, Kentaro Ueno, Ryuji Uozumi, Kentaro Ichioka, Takehiko Segawa, Tatsuo Akechi, Toshiaki A. Furukawa, Osamu Ogawa

**Affiliations:** 1 Department of Urology, Kyoto University Graduate School of Medicine, 54 Shogoinkawahara-cho, Sakyo-ku, Kyoto, 606-8507 Japan; 2 Department of Health Promotion and Human Behavior, Kyoto University School of Public Health, Kyoto, Japan; 3 Department of Psychiatry, Nagoya City University Graduate School of Medical Sciences, Nagoya, Japan; 4 Department of Urology, University of Tsukuba Hospital, Tsukuba, Japan; 5 Department of Biomedical Statistics and Bioinformatics, Kyoto University Graduate School of Medicine, Kyoto, Japan; 6 Ichioka Urological Clinic, Kyoto, Japan; 7 Department of Urology, Kyoto City Hospital, Kyoto, Japan

**Keywords:** Cognitive behavioral therapy, Overactive bladder, Randomized control trial

## Abstract

**Background:**

Overactive bladder (OAB) symptoms affect daily life by decreasing health-related quality of life (HRQol). However, there remain no very effective treatment for OAB. Pharmacotherapy is one of the best treatments, but it is not always efficient and may incur adverse events. Although behavioral therapy is another effective treatment, there are very few structured treatment manuals on how to prescribe behavioral therapy to treat OAB for whom.

Cognitive behavioral therapy (CBT) is a psychotherapy consisting of structured sessions to solve problems with the collaborative empiricism between therapists and patients. OAB symptoms are supposed to worsen with cognitive distortion, and CBT is expected to be effective in treating OAB by modifying such cognitive processes. In this trial, we will evaluate the efficacy of CBT for OAB.

**Methods:**

A randomized, controlled, open-label, multicenter parallel-group superiority trial will be conducted. Participants with moderate to severe OAB symptoms with or without pharmacotherapy will be recruited and will be randomly allocated 1:1 to two different groups by minimization (age, baseline OAB severity, treatment status, types of intervention, and treating institutions). The intervention group will be prescribed an individual CBT program covering six techniques in 4 sessions (30 min each), with or without pharmacotherapy. The primary outcome is the change scores in an OAB-questionnaire (OAB-q) from baseline to the end of the trial (week 13). Secondary outcomes will include other patient reported outcome measures and the frequency volume chart. All analyses will be conducted on an intention-to-treat principle.

**Discussion:**

This trial will determine the efficacy of CBT to treat OAB using a rigorous methodology. The effectiveness of CBT with a structured manual may not only lead to a new treatment option for patients suffering from OAB symptoms, but may also reduce the social burden by OAB.

**Trial registration:**

UMIN-CTR Clinical Trial, CTR-UMIN000038513. Registered on November 7, 2019.

## Background

Overactive bladder (OAB) is defined as “urgency, with or without urgency incontinence, usually with frequency and nocturia.” [1] The prevalence of OAB is estimated to range between 10 and 20%, and increases with age [2–4]. OAB has a negative impact on lives. Firstly, it decreases health-related quality of life (HRQoL), and the more severe the OAB symptoms, the higher the decrease [5]. Secondly, it incurs an economic burden as the estimated total cost on OAB in six Western countries was €9.7 billion [6]. To make matters worse, 43 to 83% of OAB patients discontinued pharmacotherapy within 1 month either because these were not effective or adverse events were incurred [7].

Behavioral therapy consists of lifestyle modifications, bladder training and pelvic floor muscle training (PFMT), and clinical guidelines recommend this as a first-line therapy to treat OAB because it is safe [8, 9]. A systematic review showed that behavioral therapy was more effective in treating urgent urinary incontinence (UUI) than were anticholinergics [10]. However, behavioral therapy is superseded by drug therapy. One of the reasons may be a lack of structured treatment manuals with regard to whom and how to prescribe behavioral therapy to treat OAB.

Cognitive behavioral therapy (CBT) is a psychotherapy originally developed to treat depression in 1960s [11]. CBT consists of structured sessions to address challenges through the collaborative empiricism between therapists and patients. The techniques were generalized and applied to other psychological disorders, such as panic disorder and posttraumatic stress disorder. The effectiveness CBT was demonstrated not only in the context of psychological disorders, but also in the context of functional diseases, such as irritable bowel disease [12] and chronic pain [13]. Accordingly, CBT has been increasingly applied to many diseases in addition to psychological disorders. A previous report has shown that urinary voiding is not only induced by sensation, but also by cognitive processes, and OAB is suspected to worsen as a result of cognitive distortions [14]. Therefore, we deemed that CBT may be effective in treating OAB by modifying certain cognitive processes. Previously, we had developed CBT for drug-resistant OAB and evaluated its clinical feasibility and acceptability as treatment [15].

To establish a new program to treat OAB, we will use CBT techniques and develop a structured treatment manual based on conventional behavioral therapy. In this randomized controlled trial (RCT), we will evaluate the efficacy of our new program to treat OAB.

## Methods/design

### Trial design

This study is designed as a randomized, controlled, open-label, multicenter parallel-group superiority trial. Patients will be randomly allocated 1:1 into two groups in order to evaluate the efficacy of CBT to treat OAB. The study protocol follows the Standard Protocol Items: Recommendations for Interventional Trials (SPIRIT) [16] and Template for Intervention Description and Replication (TIDieR) checklist and guide [17]. This study was approved by the Institutional Review Boards at Kyoto University Graduate School of Medicine (No. C1423) and was registered in the clinical trial registry (UMIN000035734). Written informed consent will be obtained from all participants.

### Participants

The participants will be recruited at one university hospital, two general hospitals, and one private clinic. The following inclusion criteria will be used for this trial:

1. Women between 20 to 80 years;
2. Participants not having undergone any OAB treatment or taking pharmacotherapy for OAB lasting more than 6 weeks;
3. Participants diagnosed with OAB based on their OAB symptom score (OABSS) (a total OABSS score ≥ 3, with an urgency score ≥ 2), with the total score being higher than 6 (OABSS; 6 to 11 points indicate moderate severity, 12 to 15 points indicate severity of severe nature) [18];
4. An Eastern Cooperative Oncology Group (ECOG) Performance Status of Grade 0; and
5. Participants who are able to understand explanations and sign a written informed consent.

The following exclusion criteria will be used for this trial:

1. Abnormalities around the bladder (e.g., bladder cancer, bladder calculus, interstitial cystitis, endometriosis);
2. Urinary tract infection;
3. Surgery for urinary incontinence;
4. Pregnancy;
5. Disability to understand Japanese;
6. Depression;
7. Dementia; and
8. Participants judged otherwise unsuitable for participation by the researchers. For example, participants with serious comorbidities or living far from the recruiting institution will be excluded because they are assumed to drop out from the intervention or assessment.

### Interventions

Participants will be prescribed a face-to-face, individual CBT program added onto their baseline therapy. After the start of the study, the study protocol was revised to add a treatment option via a remote teleconferencing service such as Zoom (©Zoom Video Communications, Inc) because of the spread of the coronavirus disease 2019 (COVID-19). When possible and/or necessary, sessions will be conducted via Zoom. If participants have not started or have quit drug therapy for more than 6 weeks, they will only receive CBT.

#### Development of the cognitive behavioral therapy program for overactive bladder

**Fig. 1:**
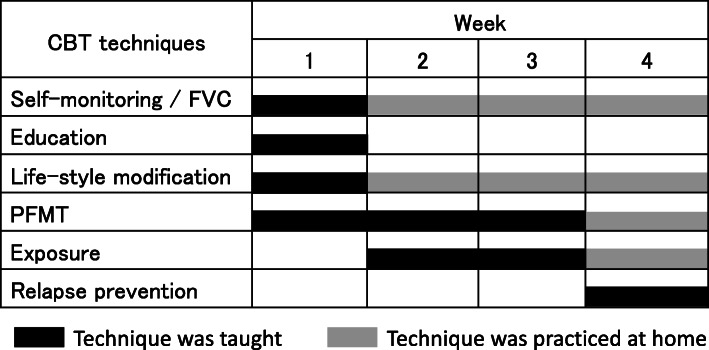
Overview of CBT for drug-resistant OAB

**Fig. 2:**
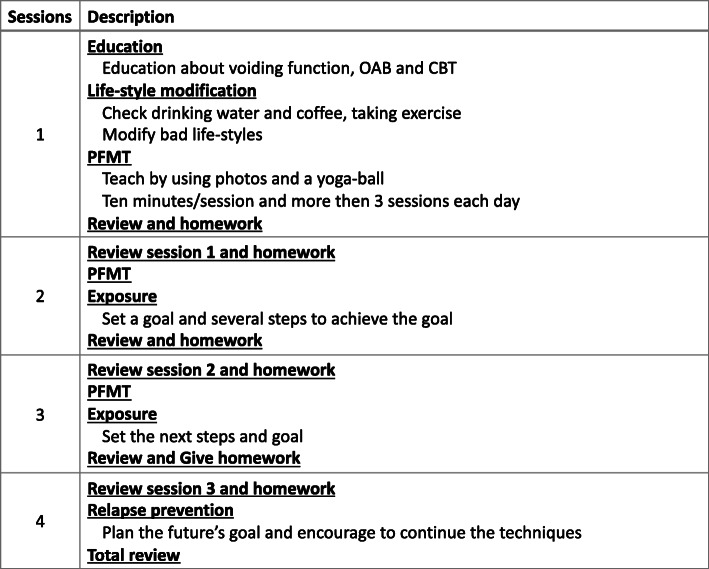
Brief summary of CBT manual for drug-resistant OAB

**Fig. 3:**
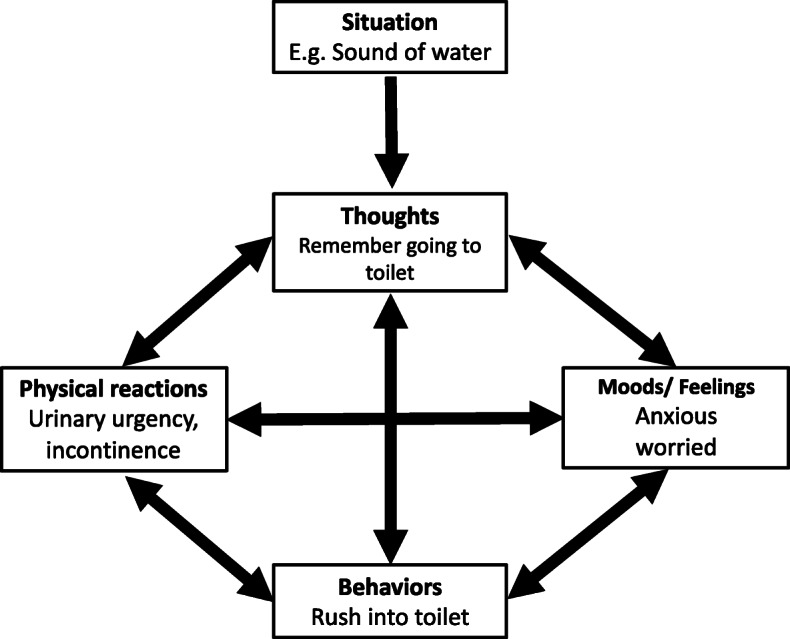
CBT model for OAB and target of each techniques

**Fig. 4:**
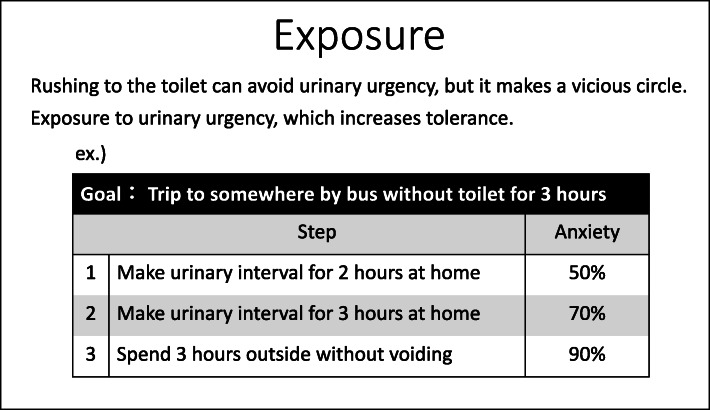
The example of the technique of exposure

The research team consists of urologists, gynecologist, psychiatrists, psychologists, and physiotherapists. We have developed a CBT program based on conventional behavioral therapy [8, 19] to treat drug-resistant OAB and CBT techniques for panic disorder, [20, 21] and evaluated the clinical feasibility and acceptability as a pilot study [15]. We simplified the CBT program to treat OAB since most participants were expected to be elderly or aging. As a result, we developed a training program based on 4 sessions (30 min each) and 6 techniques carried out over the course of 4 to 12 weeks (Fig. 1). Each session is divided into three parts, reviewing homework, providing new techniques, and reviewing the current day’s session. All sessions will be provided face-to-face, individually, at each institution or via Zoom. Brief descriptions of each of the 4 sessions are shown in Fig. 2. (1) Participants collect frequency volume charts (FVC) for self-monitoring during the intervention period. (2) Participant education consists of teaching them about the normal urinary tract system, abnormal voiding function, and epidemiology and physiology of OAB. We develop a CBT model applied to OAB and explain the targets of each CBT technique (Fig. 3). (3) Lifestyle modifications are carried out based on participants’ habits, such as by restricting water and coffee intake, as appropriate. (4) PFMT is conducted with abdominal breathing and taught by using photos and a yoga ball to enable easy imagination. (5) Exposure is modified to treat OAB based on bladder training techniques. For example, if a participant and a therapist set a goal, such as going on a bus tour for 3 h without urinary voiding, they set up several steps to achieve this goal, such as step 1: Be patient with urinary urgency and void every 2 h at home, step 2: Be patient with urinary urgency and void every 3 h at home, step 3: Be patient with urinary urgency and void every 3 h outside (Fig. 4). (6) Relapse prevention consists of planning for the future and encouraging the continuation of the taught techniques. Weekly homework is assigned in order for participants to learn the techniques and to assess participant adherence to subsequent sessions.

#### Therapist and treatment integrity

Our therapist is a urologist with 10 years of experience in urological treatment and a Japanese Board-Certified Instructor. He has attended a 2-day CBT workshop held by the Ministry of Health, Labour and Welfare of Japan. We developed a treatment manual for this study and the therapist followed the manual to ensure the reproducibility of the delivered intervention. We also attached worksheets to assist with the acquisition of each technique and homework practice. The therapist used a procedure checklist at each session to ensure adherence.

#### Waiting list control group

The control group is a waiting list. Participants will keep receiving their baseline therapy during the study period. If they are naïve or quit drug therapy for more than 6 weeks, they will not receive any therapy. After completing the trial, they will receive CBT treatment.

### Rules with regard to participant cessation

#### Deviation from protocol treatment

The following cases will be determined as deviations from the treatment protocol. However, the participants will not be determined to have dropped out of the trial at this stage and will be able to receive protocol assessments unless they withdraw consent for assessments (as below):

1. When a participant does not receive any intervention;
2. When interventions are separated by more than 3 weeks;
3. When a participant receives another treatment for OAB during the trial;
4. When there is no outcome-related data.

#### Discontinuation of the protocol treatment

The following cases will be considered as discontinuations from the treatment protocol. However, the participants will not be determined to have dropped out of the trial at this stage and will be able to receive protocol assessments unless they withdraw consent for assessments (as below):

1. If the participant wishes to stop the protocol treatment;
2. If the participant cannot continue the protocol treatment due to adverse events;
3. When the trial physician considers that the risk will be more than the benefit to continue the protocol treatment for any reason;
4. When the trial physician considers that it is not appropriate to continue the protocol treatment for any other reason.

#### Cessation of assessments

If the participant withdraws consent for assessments, he/she will not be followed up.

### Outcomes

#### Primary outcome measure

We will use the changes in HRQoL total scores of the OAB questionnaire (OAB-q) [22] from baseline to week 13 as a primary outcome in this study. The OAB-q reflects OAB-related QoL outcomes, consisting of 33 questionnaires and six subscales, assessing symptom bother, coping behaviors, concerns/worries, sleep patterns, social interaction, and total HRQoL score. Each subscale score ranges from 0 to 100, and lower symptom scores but higher scores on the other subscales reflect improvement. The minimum clinical important difference (MCID) of the OAB-q was reported to be 10 points [23].

#### Secondary outcome measures

Secondary outcomes include the following:

1. Changes in HRQoL total scores of the OAB-q at weeks 5 and 9 [22];
2. Other subscales of the OAB-q (symptom bother, coping behaviors, concern/worry, sleep and social interaction) [22];
3. King’s health questionnaire (KHQ: OAB-related QoL outcomes consist of 21 questionnaires and nine subscales [24]. Each score of the subscales ranges from 0 to 100, and lower scores indicate improvement);
4. OABSS (questionnaires to diagnose OAB and also to classify the severity [25]. The total score ranges from 0 to 15, and the severity is classified as mild (0 to 5), moderate (6 to 11), and severe (11 to 15). The MCID of the OABSS was 3 points);
5. Patient Global Impression-Improvement (PGI-I: measures patient-perceived impression of changes in symptoms with a 7-point Likert-type scale ranging from (1) “very much improved” to (7) “very much worse” [26]), which scales will be classified into 2 categories for analysis as responses (1) or (2) (“very much improved” or “much improved”) compared with all other categories;
6. Patient Global Impression-Severity (PGI-S: measures patient-perceived severity of symptoms with a 4-point Likert-type scale ranging from (1) “normal”, (2)” mild”, (3)” moderate”, or (4) “severe” [26]), which will be dichotomized into 2 categories for analysis as responses (1) or (2) (“normal” or “mild”) compared with all other categories;
7. Frequency volume chart (FVC: voiding frequency, urgency, and incontinence are documented in 3-day bladder diaries [27]);
8. Hospital Anxiety and Depression Scale (HADS: measures to assess anxiety and depression, and both scores range between 0 to 21 [28]. The status is classified as “mild” (0 to 7), “borderline” (8 to 10), and “severe” (11 to 21));
9. Five-level EuroQol five-dimensional questionnaire (EQ-5D-5L: health status is assessed with 5 items, 5 subscales [29]);
10. Treatment satisfaction (5 points Likert scale questions, “How is your satisfaction with your treatment?”; (1) “very satisfied,” (2) “satisfied,” (3) “neutral,” (4) “dissatisfied,” and (5) “very dissatisfied”), which will be dichotomized into 2 categories for analysis as responses (1) or (2) (“very satisfied” or “satisfied”) compared with all other categories;
11. Drop-out rate;
12. Change in pharmacotherapy (“Did you change your medication during the treatment period?”; (1) “no change,” (2) “add medication,” (3) “decrease medication,” or (4) “quit medication”);
13. Adherence (evaluated as the week rate of homework completion).

#### Baseline characteristics measures

Participants will complete a form on demographics including age, height, weight, comorbidity, medicine, delivery, menopause, education, marital and job status, and history of OAB treatment.

#### Participant timeline

**Fig. 5:**
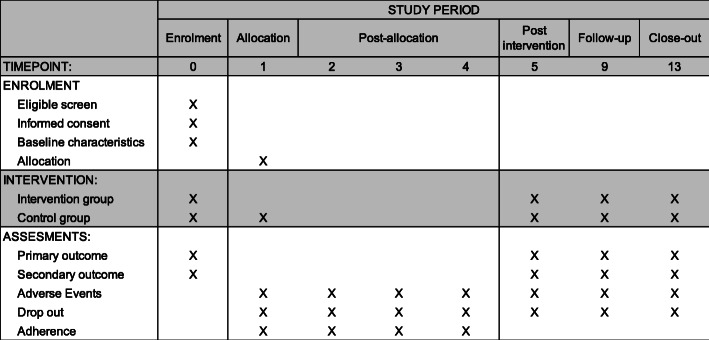
The schedule of this study

Fig. 5 shows the participant timeline reflecting enrollment, intervention, and assessment. Enrollment will be carried out at each institution. Following screening, the trial will be explained to eligible participants. After completing informed consent, participants will fill out the questionnaires about baseline characteristics. Participants will complete a self-report questionnaire in a written paper format or via electronic data capture at baseline (week 0), immediately after intervention (week 5), and at follow-up (week 9 and week 13)).

#### Sample size

The primary outcome is change from baseline to week 13 in HRQoL total scores of the OAB-q between groups. A total of 128 participants are required to detect a moderate effect size of 0.5 between the groups based on a two-sided significance level of 0.05 and 80% power [30]. Assuming a 15% drop out rate based on a previous RCT to evaluate behavioral therapy in women with mixed urinary incontinence [31], we set the target sample size at 150 participants.

#### Recruitment

General hospitals and private clinic providers will identify potential participants during clinic visits. Screening interviews will be carried out to assess participant eligibility. When possible and/or necessary, the recruitment and screening interview will be conducted via Zoom. We are planning to recruit participants within two and a half years from trial start.

### Assignment of interventions

#### Sequence generation

The random allocation will be carried out by UMIN INDICE (UMIN Internet Data and Information Center of Clinical Research, https://www.umin.ac.jp/indice/cloud.html) cloud, an internet-based central randomization cloud system. A minimization algorithm will be used to ensure balance across age (20- to 64-year-olds vs 65- to 80-year-olds), baseline OAB severity (moderate (OABSS: 6 to 11) vs severe (OABSS: 11 to 15)), treatment status (no treatment vs past treatment vs under treatment), types of intervention (face-to-face vs online remote session), and treating institutions.

#### Allocation concealment and implementation

In week 1, the therapist will access the UMIN INDICE cloud in front of participants. The random allocation to either group at a 1:1 ratio will be centrally generated via the UMIN INDICE cloud after the necessary baseline information is entered, and opened at the same time to the therapist. Allocation concealment will therefore be ensured. Immediately after this, the intervention group will be prescribed CBT, and the waiting list control group will be informed of the schedule.

#### Blinding

In this open label study, participants and a therapist will be aware of the intervention assigned. Not all outcome assessments will be blinded due to the nature of the patient reported outcome measures (PROM). The statistician will be blinded to the allocation during the analyses.

### Data collection

#### Data collection methods

Outcome data will be collected at baseline (week 0), immediately after intervention (week 5), and at follow-up (week 9 and week 13) using self-reported questionnaires in paper form or via electronic data capture.

#### Data management

Outcome data will be transferred into a Research Electronic Data Capture (REDcap) system (https://www.project-redcap.org/). The therapist will enter the data on baseline characteristics, allocation, drop out, changes of pharmacotherapy, homework adherence, and adverse events into REDcap. If any missing data is detected via the REDcap system, the researcher will contact the participants by telephone.

#### Statistical methods

All analyses will be carried out based on an intention-to-treat principal, considering all participants as randomized, regardless of whether they received the randomized treatment. We do not plan to conduct any interim analyses.

#### Analysis of primary outcome

We will use a linear mixed-effects model with repeated-measures analysis to estimate the least-squares mean difference in score changes from baseline at weeks 5, 9, and 13 in the HRQoL of the OAB-q between group comparisons. This model assumes that primary outcome data are missing at random and includes study group (intervention and control), assessment points (week 5, week 9, and week 13), OAB severity (moderate and severe), baseline score, and the interaction of study group with assessment points. Week 13 is the assessment point for the primary analysis. A multiple-testing procedure will be used to control for the familywise type I error rate at a two-sided significance level of 0.05. If the primary analysis at week 13 shows statistical significance, testing of other assessment points of the primary outcome is to be performed in a hierarchical manner: week 9 and week 5.

#### Analysis of secondary outcomes

Secondary outcomes are important in order to measure the wider effects for participants, and will be similarly analyzed (as appropriate for continuous or dichotomous outcomes). No adjustments for multiplicity are made for the secondary outcomes except the HRQoL total scores of the OAB-q, as shown above. Thus, point estimates and 95% confidence intervals will be reported, without *P* values. The 95% confidence intervals are not adjusted for multiple comparisons, and inference drawn from these may not be reproducible. These analyses will be exploratory in nature in order to complement the primary analyses.

#### Adverse events

We will convey the numbers and frequencies (%) of adverse events.

#### Subgroup analyses

We will perform the following subgroup analyses:

1. Participants: aged 20 to 64 years vs 65 to 80 years;
2. OAB severity: moderate (OABSS: 6 to 11 points) vs severe (OABSS: 12 to 15 points);
3. Pharmacotherapy: naïve vs past vs under treatment;
4. Incontinence: no (OABSS Q4 = 0 point) vs yes (OABSS Q4 = 1 to 5 point);
5. HADS Anxiety score: normal (0 to 7 points) vs borderline or abnormal (8 to 21 points)
6. HADS Depression score: normal (0 to 7 points) vs borderline or abnormal (8 to 21 points).

When possible and/or necessary,

7) Intervention type: face-to-face vs online remote session

#### Sensitivity analyses

Participants who (1) receive all intervention treatment; (2) have had intervention intervals lasting less than 3 weeks; (3) have not received any other treatment for OAB; and (4) have generated full outcome data will be analyzed as per protocol set (PPS). Furthermore, sensitivity analyses including multiple imputation approach for imputing missing data will be performed, with missing-at-random assumptions.

### Monitoring

#### Data monitoring

There was no composition of data monitoring committee no interim analysis in this study.

### Reporting of adverse events and protection of participants

#### Definition of adverse events

An adverse event is defined as any unintended or unwanted symptom, sign, or disease seen in participants. The Japanese Ministry of Health, Labor and Welfare publish, “Ethical Guidelines for Clinical Studies: Questions and Answers,” and it defines severe adverse events as following: a) death; b) threatened death; c) admission or prolongation of admission for treatment; d) enduring and severe impairment and dysfunction; or e) congenital anomaly.

When such a serious adverse event will happened, the trial physician must inform the principal investigator within 48 h, with or without the causal relationship to the protocol treatment. The principal investigator will report to the Institutional Review Boards in Kyoto University Graduate School of Medicine within 72 h, also inform all collaborators at all participating institutions. If it occurs an unexpected serious adverse event, the principal investigator will report it to the Ministry of Health, Labor and Welfare.

#### Auditing

Any formal audits will not be performed, because this study’s intervention can be classified as a “minimally invasive intervention”.

### Ethics and dissemination

#### Research ethics approval

This study protocol was approved by the Institutional Review Boards of Kyoto University Graduate School of Medicine (No. C1457) on January 15, 2020. The protocol was revised to add a treatment option via Zoom because of the spread of the coronavirus disease 2019 (COVID-19) and re-approved on June 30, 2020.

#### Protocol amendments

The steering committee will determine any revisions of the protocol. Any amendments of the protocol will be submitted to the Institutional Review Boards of Kyoto University Graduate School of Medicine for approval. The amended protocol will also be submitted to the Institutional Review Boards of the other participating institutions and reported to the participants as necessary.

#### Consent or assent

Informed consent will be obtained at each institution. Following the screening, the trial will be explained to eligible participants. Next, researchers will obtain full informed written consent from participants.

#### Confidentiality

An identification number will be assigned to each participant on enrollment. This number will be used for data registration. The correspondence list is kept securely in the computer on which the password has been set. After completion of the trial, the raw data will be kept in a locked drawer at the Kyoto University Graduate School of Medicine for 10 years following the publication of the first survey results.

#### Access to date

The final trial dataset will be able to access to all members of the steering committee and the statistician. After the dataset will be de-identified and anonymized, it will be uploaded to the UMIN-ICDR website (http://www.umin.ac.jp/icdr/index-j.html), and researchers who will be approved by the steering committee will be able to access to the dataset.

#### Ancillary and post-trial care

Because all protocol interventions are administered within the scope of the approved regulations in Japan, any health hazards will be covered by Japan’s National Health Insurance.

#### Dissemination policy

The study results will be disseminated to the public in academic conferences and journals. The steering committee will determine authorship of the planned primary and secondary publications. The order of the authors will be decided by the contributions of each member. The authors of the paper and the conference presenters will be based on the Uniform Requirements for Manuscripts Submitted to Biomedical Journals of the International Committee of Medical Journal Editors.

## Discussion

We have reported the protocol for a randomized, controlled, open-label, multicenter superiority trial seeking to evaluate the efficacy of CBT for OAB. To our knowledge, this is the first trial investigating the efficacy of CBT for OAB in a clinical setting. We have previously developed a CBT program for drug-resistant OAB and evaluated the clinical feasibility and acceptability of the treatment in a pilot study [15]. We will rigorously evaluate the efficacy of CBT for OAB in this RCT.

There are several limitations to this study. First, this will be an open-label designed RCT, with unblinded participants, therapist, and outcome assessors. The primary outcome, OAB-q, is a PRO questionnaire, and the unblinding will result in a certain degree of assessment bias, which may favor the intervention group. Although we have considered relatively hard outcomes, such as voiding frequency, the purpose of treatment is to improve patients’ QOL, to which end we have selected OAB-q as the primary outcome. Secondly, although this treatment will be administered using a manual, it will be conducted by a urologist, meaning that its general applicability cannot be fully ascertained. To improve generalizability, more therapists will be needed to participate in further evaluations of this approach.

This study has some critical implications for clinicians and patients. This new approach is expected to treat OAB with few adverse events compared with drug therapy. Moreover, this CBT treatment consists of a structured manual that enables therapists of various backgrounds to administer the therapy in an efficient, consistent way. As it may be applied to group therapy, or smartphone therapy, more and more patients may eventually benefit from this CBT approach. It may solve the issue of the shortage of CBT therapists and increasing the medical cost.

## Data Availability

Researchers approved by the steering committee will be able to have access to the dataset. After publishing the primary findings, the de-identified and completely anonymized individual participant-level dataset will be posted on the UMIN-ICDR website (http://www.umin.ac.jp/icdr/index-j.html) for access by qualified researchers.

## Abbreviations

CBT: Cognitive behavioral therapy
FVC: Frequency volume chart
HRQoL: Health-related quality of life
MCID: Minimum clinical important difference
OAB: Overactive bladder
OABSS: OAB symptom score
PFMT: Pelvic floor muscle training
PPS: Per protocol set
RCT: Randomized controlled trial
UUI: Urgent urinary incontinence
